# Transcatheter aortic valve implantation (TAVI) planning CT on 8‐cm detector scanners: Proper dose control by combined use of two deep‐learning reconstruction algorithms

**DOI:** 10.1002/acm2.70224

**Published:** 2025-08-29

**Authors:** Qiang Shao, Yongxia Zhou, Jing Li, Guozhi Zhang, Kunyao Li, Zhaojun Ding, Rong Zhou, Jiamo Zhang, Xiaohong Zheng, Yonghong Du

**Affiliations:** ^1^ State Key Laboratory of Ultrasound in Medicine and Engineering, College of Biomedical Engineering Chongqing Medical University Chongqing China; ^2^ Department of Radiology Yongchuan Hospital of Chongqing Medical University Chongqing China; ^3^ United Imaging Healthcare Shanghai China; ^4^ Chongqing Medical and Pharmaceutical College Chongqing China

**Keywords:** computed tomography angiography, deep learning reconstruction, radiation dose, transcatheter aortic valve implantation

## Abstract

**Purpose:**

To explore the feasibility of transcatheter aortic valve implantation (TAVI) planning computed tomography (CT) on single‐source 8‐cm detector scanners with proper dose control by using two deep‐learning reconstruction algorithms.

**Methods:**

Reduced‐dose TAVI planning CT was simulated by replacing routine aortic CT angiography (CTA) with a reduced‐dose aortic CTA and a reduced‐dose coronary CTA (Group A, *n* = 82), while keeping the total dose unchanged. Each of the two CTA scans was processed with a different deep‐learning reconstruction algorithm. Routine‐dose coronary CTA (Group B, *n* = 86) and routine‐dose aortic CTA (Group C, *n* = 77) with hybrid iterative reconstruction were used as reference for evaluating the acceptability for surgical planning (A vs. B for aortic valve; A vs. C for access route) and for comparing both the diagnostic and objective image quality (A vs. B for coronary arteries; A vs. C for aortoiliac arteries).

**Results:**

The mean effective dose in Group A was 8.22 ± 0.83 mSv, representing a 57% reduction of the routine‐dose TAVI planning CT, that is, a routine‐dose coronary CTA plus a routine‐dose aortic CTA on the same scanner model. With respect to B and C, images in A were scored higher for evaluating the aortic valve (*p *= 0.045) and the access route (*p *= 0.014) and for diagnosing the thoracic aorta and iliac segments (*p *< 0.050), while the diagnostic confidence were comparable on the coronary arteries (*p *> 0.050), abdominal aorta (*p *= 0.276), and femoral segment (*p *= 0.816). The image noise in A was found to be 21%–55% lower, leading to a significant increase in contrast‐to‐noise ratio (CNR) by 63%–114% (*p *< 0.050).

**Conclusion:**

Reduced‐dose TAVI planning CT is feasible on 8‐cm detector scanners by using deep‐learning reconstruction algorithms, showing promise of implementing the examination in imaging settings that are more commonly accessible.

## 1 INTRODUCTION

Transcatheter aortic valve implantation (TAVI) is a minimally invasive procedure to tackle severe symptomatic aortic stenosis, which has been proven with almost equivalent efficacy to conventional surgical aortic valve replacement.^1^, ^2^, ^3^, ^4^ Pre‐procedural work‐up plays a crucial role in patient selection as well as in evaluating the peripheral access route and the prosthesis deployment. In this context, computed tomography (CT) is an advantageous choice for its ability to provide comprehensive information on the entire vasculature of aortic‐iliac‐femoral arteries and on the coronary arteries within a single contrast‐enhanced angiographic examination.^5^, ^6^, ^7^ However, TAVI planning CT does involve a long scan coverage that consists of a coronary CT angiography (CCTA) and an aortoiliac CT angiography (CTA), resulting in a significant radiation dose.^8^ Proper dose control while ensuring the required image quality, therefore, remains an ongoing topic in current investigations of TAVI planning CT.

Early studies have shown the value of low tube voltage in reducing the dose of TAVI planning CT.^9^, ^10^ Later on, ultra‐high pitch acquisition with dual‐source CT scanners was introduced to TAVI planning CT, which managed to reduce the radiation to as low as around 3.0 mSv.^11^, ^12^ However, the prospectively electrocardiogram‐gated (ECG‐gated) CCTA on a dual‐source CT dose places certain restrictions on the patient's heart rate. In comparison, the TAVI planning CT protocols implemented on wide‐detector CT scanners, with an iconic size of 16 cm, are normally considered more capable of handling patients with an exceeding heart rate.^13^, ^14^ On radiation dose, the emerging deep‐learning‐based CT image reconstruction algorithms seem to be a promising solution on such scanners, as demonstrated in the work of Zhang et al.,^15^ where the dose was below 5 mSv.

Nowadays, very few TAVI planning CTs are conducted on 4‐ to 8‐cm detector CT scanners, as opposed to those with dual source or wide detectors. One of the main reasons might be the associated radiation dose, which arises substantially due to the retrospective ECG‐gated CCTA acquisition in helical mode. The total dose of one typical TAVI planning CT may go as high as 20 mSv.^16^ To the best of our knowledge, dose reduction measures such as using the deep‐learning reconstruction algorithm have not been investigated for TAVI planning CT on scanner models of this tier. In other words, if the dose issue gets sufficiently addressed, the requirement for implementing the TAVI planning CT would be lowered to a common imaging setting, which is far more available and accessible to potential TAVI patients. With the growing applicability of TAVI, accordingly, a considerable clinical impact can be expected.

The purpose of this study was to test the hypothesis that deep‐learning reconstruction algorithms allow TAVI planning CT to be carried out at a reduced dose with excellent imaging performance and to explore the feasibility of reduced‐dose TAVI planning CT on a single‐source 8‐cm detector CT scanner model with combined use of two deep‐learning reconstruction algorithms. Not only was it the first study focusing on low‐dose TAVI planning CT on such scanner models, but it was also the first to make use of two different advanced deep‐learning reconstruction algorithms for the same purpose in one specific application. Additionally, given the limited number of cases on this scanner at the present time, it was proposed in this study to simulate an equivalent scenario of TAVI planning CT with patients undergoing a different CT examination.

## 2 MATERIALS AND METHODS

### 2.1 Study design and patient population

**FIGURE 1:**
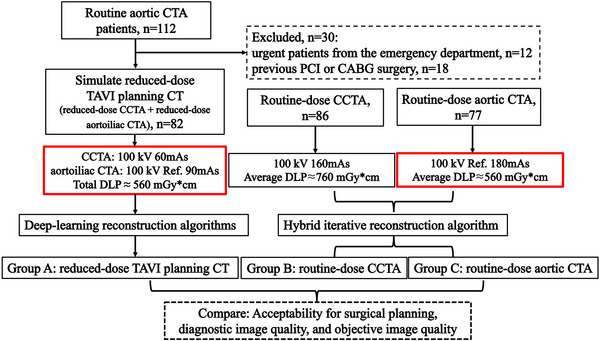
Flowchart of the study design. CABG, coronary artery bypass graft; CCTA, coronary CT angiography; CT, computed tomography; DLP, dose‐length product; PCI, percutaneous coronary revascularization; TAVI, transcatheter aortic valve implantation.

This prospective study was approved by the Institutional Review Board (2024EC0005). As illustrated in Figure 1, patients scheduled for routine aortic CTA were enrolled upon informed consent to simulate a scenario of TAVI planning CT at a reduced dose. The design was to lower the dose in aortic CTA and add a CCTA, where the dose in total was kept the same as in the original aortic CTA examination. It is worth mentioning that the longitudinal scan range of a routine aortic CTA is similar to that of the aortoiliac CTA in TAVI planning CT for most cases, so the two terms are considered interchangeable in this context. Given the inherent correlation among cardiovascular diseases, those who choose to participate would benefit from the additional CCTA without having to bear an extra radiation dose. The study was conducted continuously from December 2023 to March 2024. Patients presenting acute symptoms that were suspected of aortic dissection or aortic aneurysm rupture were not included due to the potentially urgent nature of these cases. Also excluded were those with cardiovascular complications where the dose was a less important issue.

Since there have not been sufficient cases of real TAVI planning CT conducted on this 8‐cm detector CT scanner, the collected data of the simulation scan (Group A) were compared against retrospectively collected routine‐dose CCTA data (Group B) and routine‐dose aortic CTA data (Group C), from May to November 2023, where each corresponding part, for example, the aortic valve, access route, and the coronary arteries, was assessed in the way required for TAVI planning in practice, including both diagnostic and quantitative measures. In collecting the data of groups B and C, the same exclusion criteria as A were applied to ensure the comparison was set on a similar baseline.

### 2.2 CT image acquisition

All simulation and retrospective images were acquired on a 160‐row 8‐cm detector CT scanner (uCT 860, United Imaging Healthcare, Shanghai, China). The simulated TAVI planning CT consisted of one CCTA, ranging from the tracheal bifurcation to the bottom of the heart, and one aortoiliac CTA, ranging from the mandibular angle to the femoral heads, which were combined in one examination with one administration of contrast media. The exposure parameters were adapted from both the routine CCTA scan: retrospective ECG‐gated, 80 mm collimation, automatic pitch depending on the patient's heart rate, 0.25 s rotation time, 100 kVp, and 160 mAs, and the aortic CTA scan: non‐gated, 80 mm collimation, 0.99 pitch, 0.25 s rotation time, 100 kVp, and reference tube current 180 mAs with automated tube current modulation. Specifically, the reference tube current for the simulated TAVI planning CT on aortoiliac arteries was set to 90 mAs, which was half of that in routine aortic CTA, and the reduced amount of dose, in terms of dose‐length product (DLP), was mapped to the scan on coronary arteries, where the tube current was determined to be 60 mAs with respect to the DLP of routine CCTA. In other words, the simulation of TAVI planning CT at reduced dose was realized by splitting the dose in routine aortic CTA and transferring half of it to the added CCTA.

All patients were applied a biphasic contrast administration protocol with an initial injection of 55 mL of undiluted contrast agent (Iopamidol, concentration 370 mgI mL^−1^) at a flow rate of 5.0 mL s^−1^, followed by 50 mL of diluted contrast material (1:1 contrast material to saline) injected at the same flow rate. Bolus‐tracking was implemented via a region of interest (ROI) placed on the descending aorta with a triggering threshold of 120 HU after a delay time of 6 s. It is worth mentioning that the dosage of contrast medium used for simulating the TAVI planning CT went slightly over (10 mL) that in the routine aortic CTA protocol, which was 70 mL in total with a different injection rate (4 mL s^−1^), triggering threshold (170 HU), and delay time (10 s).

The effective dose (ED) was calculated by summing the DLP of the CCTA multiplied by a region‐specific conversion coefficient of 0.014 mSv mGy*cm^−1^ and that of the aortoiliac CTA with 0.015 mSv mGy*cm^−1^.^15^

### 2.3 CT image reconstruction

CCTA and aortic CTA images in groups B and C were reconstructed with the routine hybrid iterative algorithm (Karl 3D, United Imaging Healthcare, Shanghai, China). For group A, a deep learning trained algorithm (DELTA) was employed for the CCTA images, and a deep‐learning‐based so‐called artificial intelligence iterative reconstruction (AIIR) algorithm was employed for the aortoiliac CTA images. Both algorithms were developed by the same vendor (United Imaging Healthcare, Shanghai, China).

Specifically, DELTA, currently marketed with the name CardioBoost, makes use of a convolutional neural network (CNN) trained on over 100 000 paired images—standard‐dose filtered back projection (FBP) images and corresponding simulated low‐dose images—to suppress noise and enhance contrast. The network is further optimized to selectively improve the visibility of critical anatomical features such as plaques, stents, and vessel contours.^17^ On the other side, AIIR is a CT reconstruction algorithm that integrates a dedicated CNN into the model‐based iterative reconstruction (MBIR) framework. It replaces the traditional regularization term in MBIR—typically used to suppress noise and artifacts—with a CNN trained on large datasets consisting of paired standard‐dose (noise‐free) and simulated low‐dose (noisy) images. This integration leverages the strengths of MBIR in preserving image detail and the CNN's capability to effectively reduce image noise and maintain texture fidelity.^18^ Though investigated on scanner models that were different than the one in this study, both algorithms have demonstrated remarkable value in a variety of clinical and experimental applications.^19^, ^20^, ^21^, ^22^, ^23^, ^24^, ^25^

In obtaining the CCTA images, for both groups A and B, the same automated phase selection (ePhase, United Imaging Healthcare, Shanghai, China) and AI‐based cardiac motion correction (CardioCapture, United Imaging Healthcare, Shanghai, China) were applied.

All reconstructions used a 512 × 512‐pixel matrix, where a 0.5 mm slice thickness/interval was set for CCTA images and a 1.0 mm slice thickness/interval for all aortic and aortoiliac CTA images.

### 2.4 Analysis of acceptability for surgical planning

The acceptability of the resulting image quality for TAVI planning, with a focus on morphological and access route evaluation, was assessed by two radiologists with 8 years (Z.D.) and 10 years (K.L.) of experience relating to TAVI surgery, respectively. The image quality of the aortic valve, including the depiction of aortic valve tips, leaflet nadirs, and aortic annular contours, was graded for groups A and B using a 5‐point Likert scale (1 = poor; 5 = excellent).^15^, ^26^ Similarly, that of the access route, including the depiction of the entire vasculature and the vascular tortuosity, was graded for groups A and C using another 5‐point Likert scale (1 = poor; 5 = excellent).^9^

The grading was carried out with the readers being blinded to the patient demographics and reconstruction algorithms. In each of the two sessions, cases were mixed and presented in random order. After independent grading, the average score of two readers for each case was taken as its final score, where scores ≥3 were considered clinically acceptable.

### 2.5 Analysis of diagnostic image quality

The resulting image quality for diagnosing coronary and aortoiliac arteries, which was also a crucial part of TAVI planning CT, was assessed by another two radiologists with 15 years (J.Z.) and 9 years (R.Z.) in cardiovascular imaging, respectively. Three main coronary arteries were graded separately for groups A and B: the left anterior descending (LAD), the left circumflex (LCX), and the right coronary artery (RCA). Four segments of the aortoiliac artery were graded for groups A and C: the thoracic aorta, the abdominal aorta, the iliac artery, and the femoral artery. One same 5‐point Likert scale in terms of diagnostic confidence was employed, focusing on the delineation of vessels and detectability of plaques or stenosis involved therein (1 = poor; 5 = excellent). The average score of two readers for each vessel or segment was taken as its final score. The same blinding, randomization, and acceptance threshold as in the analysis acceptability for surgical planning was used. Additionally, a two‐category sub‐analysis based on the identified stenosis severity (<50% or ≥50%) was conducted for the scores of diagnostic image quality obtained on coronary arteries for groups A and B.

### 2.6 Analysis of objective image quality

All quantitative measurements were performed by one radiologist with over 15 years (Y.Z.) of experience in cardiovascular imaging. The mean CT number and the noise, represented by the standard deviation (SD), were measured for groups A and B on the aortic root, LAD, LCX, and RCA. The ROI size on the aortic root was set to 2/3 of the lumen, and those on coronary artery branches were 2 mm^2^. An additional measurement was performed on the pericardial fat with an ROI size of 12 mm^2^, for obtaining the contrast‐to‐noise ratio (CNR) with CNR = (𝜇_artery_−𝜇_fat_)/𝜎_fat_, where 𝜇 and 𝜎 stand for the mean of the CT number and the SD within the ROI, respectively, and the subscript ‘artery’ denotes the aortic root or the coronary arteries and ‘fat’ denotes the pericardial fat. All ROIs were placed avoiding the vessel wall or any possible calcification or plaque. In cases of severe calcifications or motion artifacts, the involved segment was excluded from evaluation.

The same was measured for groups A and C at three positions on the thoracic aorta with an ROI of 150 mm^2^: the ascending aorta, the aortic arch, and the descending aorta at the level of the pulmonary trunk and diaphragm; at two positions on the abdominal aorta with an ROI of 100 mm^2^: the level of the renal arteries and right above the bifurcation; and at three positions on the iliofemoral arteries: the common iliac arteries (ROI 30 mm^2^), the external iliac artery (ROI 30 mm^2^), and the common femoral arteries (ROI 20 mm^2^), where the average of the left and right sides was taken for the common iliac arteries and for the common femoral arteries. Similarly, an additional measurement was performed on the erector spinae muscle (ROI 80 mm^2^) for obtaining the CNR with CNR = (𝜇_artery_−𝜇_muscle_)/𝜎_muscle_.

### 2.7 Statistical analysis

All the statistical analyses were performed with SPSS version 22 (IBM Corp., Armonk, New York, USA). Normality of data was tested using the Kolmogorov–Smirnov test. Normally distributed data were compared using the *t*‐test (*n* = 2) or the Kruskal–Wallis H test (*n* > 2), while non‐normally distributed data were compared using the Mann‐Whitney U test (*n* = 2) or the Kruskal–Wallis test (*n* > 2). A *p* < 0.05 was considered statistically significant. The Cohen's kappa test was used to evaluate the inter‐observer consistency: κ of 0.81–1.0, 0.61–0.80, 0.41–0.60, 0.21–0.40, and ≤0.20 were considered excellent, good, moderate, fair, and poor agreement, respectively.

## 3 RESULTS

### 3.1 Patient characteristics and radiation dose

A total of 245 patients were enrolled for investigation, with patient characteristics and radiation dose information summarized in Table 1. Groups A, B, and C included 82, 86, and 77 patients, respectively. There was no statistically significant difference in age (*p* = 0.816) or body mass index (*p* = 0.328) among the three groups of patients. The patient's heart rate at the examination in group A was not statistically different from that in group B (*p* = 0.407). In group A, the DLP of reduced‐dose CCTA was reduced by 60% compared to group B, and the reduced‐dose aortoiliac CTA showed a reduction of 51% compared to group C. The average effective dose in group A represents a 57% reduction to that in groups B plus C, that is, routine‐dose TAVI planning CT.

**TABLE 1:** Patient characteristics and radiation dose.

	Group A Reduced‐dose TAVI planning CT	Group B Routine‐dose CCTA	Group C Routine‐dose aortic CTA	*p*
Gender (female/male)	*n* = 82 (49/33)	*n* = 86 (43/43)	*n* = 77 (47/30)	
Age, year	61.52 ± 9.25	62.40 ± 10.69	61.58 ± 11.05	0.816
BMI, kg m^−2^	24.72 ± 4.06	24.13 ± 3.59	23.68 ± 3.53	0.328
HR, bpm	72.70 ± 12.73	75.01 ± 14.23	—	0.407
Clinical history				
Hypertension	24 (29%)	31 (36%)	18 (23%)	
Type II diabetes	12 (15%)	17 (20%)	13 (17%)	
Hyperlipidemia	16 (20%)	27 (31%)	6 (8%)	
Stenosis severity				
Stenosis <50%	37 (15%)	61 (24%)	—	
Stenosis ≥50%	34 (14%)	30 (12%)		
DLP, mGy*cm	CCTA: 296.25 ± 50.59	761.65 ± 213.91	549.03 ± 72.57	
	Aortoiliac CTA: 271.40 ± 36.57			
	Total: 567.65 ± 58.18			
ED, mSv	Total: 8.22 ± 0.83	10.66 ± 2.99	8.24 ± 1.09	

*Note*: Data are presented as mean ± standard deviation, number, and frequencies in parentheses.

Abbreviations: BMI, body mass index; CCTA, coronary CT angiography; CT, computed tomography; CTA, CTA, CT angiography; DLP, dose‐length product; ED, effective dose; HR, heart rate; TAVI, transcatheter aortic valve implantation.

### 3.2 Acceptability for surgical planning

**FIGURE 2:**
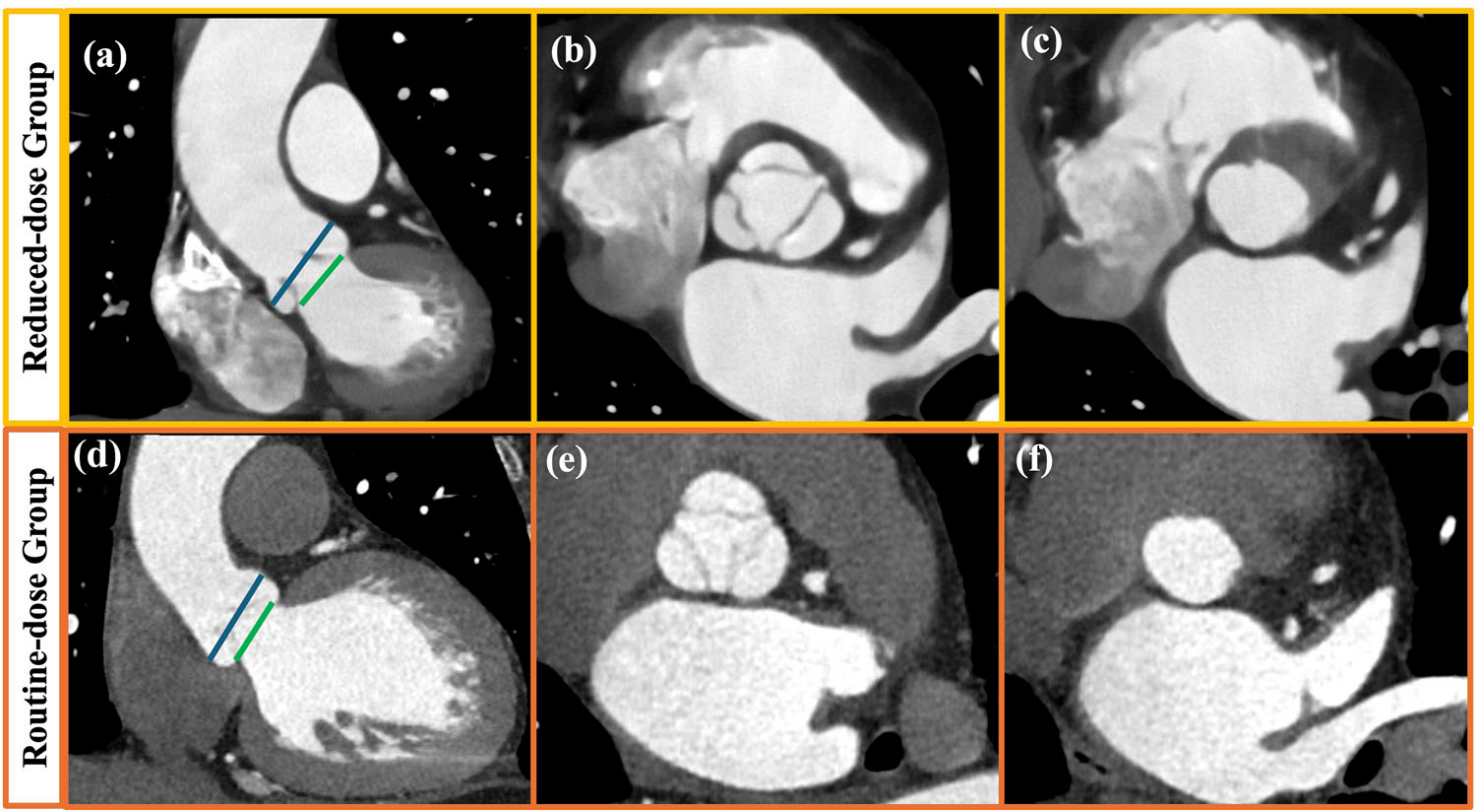
Evaluation of the aortic valve and aortic annulus on CT images. (a) and (d): TAVI assessment of valvular cusp (blue line) and aortic annulus levels (green line) in the coronal view; (b) and (e): aortic valve images at the valvular cusp level; (c) and (f): aortic valve images at the aortic annulus level. Upper row: the coronal and axial CTA images of a 69‐year‐old man (BMI, 21 kg m^−2^, HR, 77 bpm) in group A; lower row: the coronal and axial CTA images of a 59‐year‐old man (BMI, 22 kg m^−2^, HR, 68 bpm) in group B (WW/WL = 800/200 HU). Group A: reduced‐dose TAVI planning CT; Group B: routine‐dose CCTA; BMI, body mass index; CCTA, coronary CT angiography; CT, computed tomography; CTA, CT angiography; HR, heart rate; TAVI, transcatheter aortic valve implantation.

**FIGURE 3:**
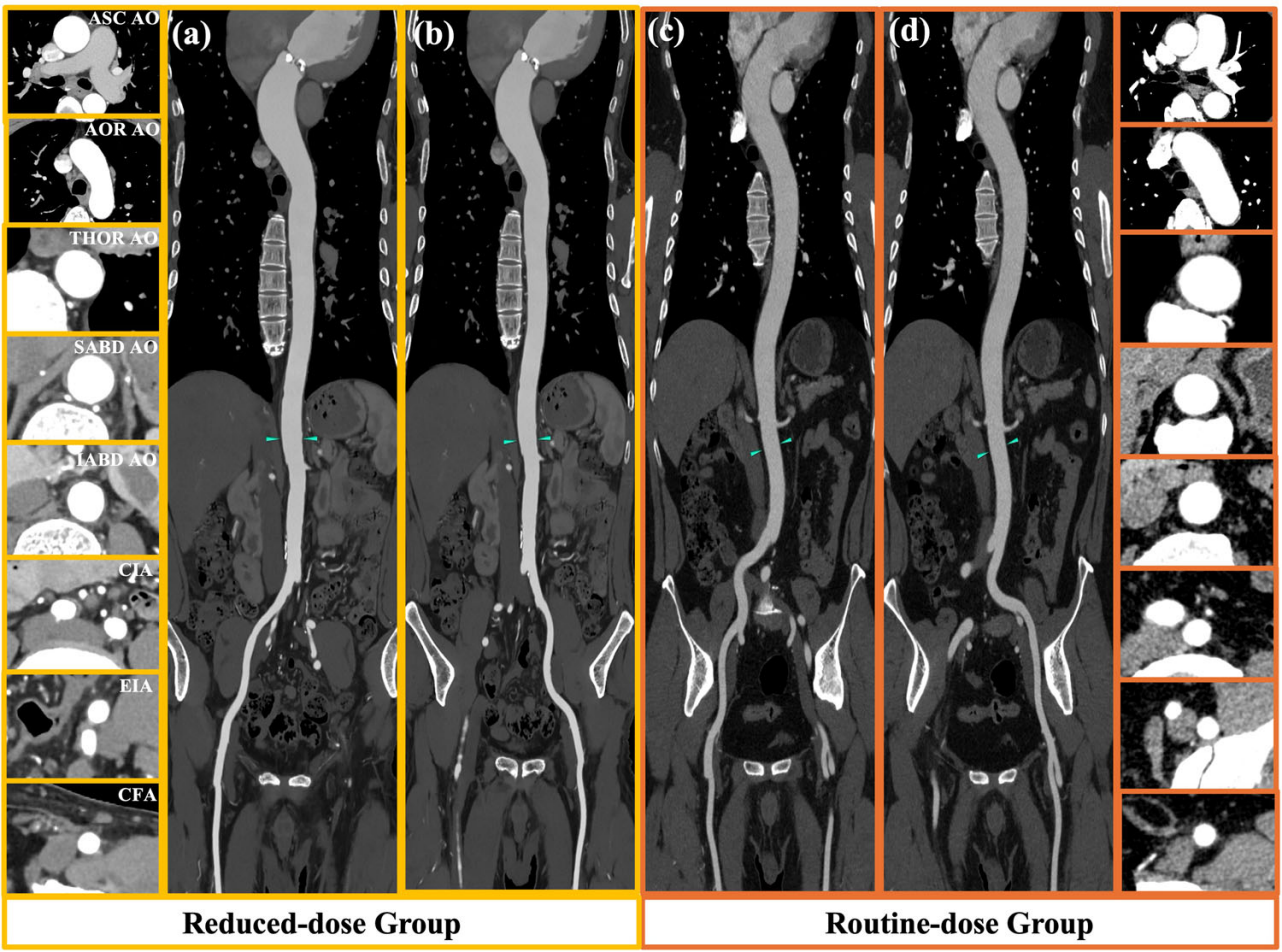
CT images of the aortic‐iliac‐femoral vasculature illustrating the access route with enlarged axial views of different sites. The display window level setting is WW/WL = 350/40 HU. (a) and (c) represent the right aortoiliac artery access route, and (b) and (d) represent the left aortoiliac artery access route. AOR A, aortic arch; ASC AO, ascending aorta; CFA, common femoral artery; CIA, common iliac artery; CT, computed tomography; EIA, external iliac artery; IABD AO, infrarenal abdominal aorta; SABD AO, suprarenal abdominal aorta; THOR AO, thoracic aorta.

Figure 2 shows the images of the aortic valve and the aortic annulus for one typical case in group A and compares them to one typical case in group B, where the view is set on planes useful for morphological evaluation in TAVI planning. The visualization of the aortoiliac vasculature is compared between groups A and C in Figure 3. The mean acceptability scores by the two radiologists as well as the inter‐observer agreement are listed in Table 2. Group A received higher scores on the aortic valve than group B (*p* = 0.045), where the DELTA reconstruction has noticeably improved the contrast resolution around the valvular cusp even at reduced dose, as illustrated in Figure 2. For assessing the access route, all cases were scored ≥3 in both groups, suggesting adequate acceptability for TAVI planning. Nonetheless, the mean score for group A was statistically higher than that for group C (*p* = 0.001). Although all inter‐observer agreement was moderate and above (0.70 ≤ κ ≤ 0.89), the two readers were more aligned when grading the images of the aortic valve, where the difference between the reduced‐dose TAVI planning CT by deep‐learning reconstruction and the routine CCTA was more obvious.

**TABLE 2:** Scores of acceptability for TAVI planning.

	Group A Reduced‐dose TAVI planning CT		Group B Routine‐dose CCTA		Group C Routine‐dose aortic CTA		*p*
	Score (mean ± SD)	Kappa	Score (mean ± SD)	Kappa	Score (mean ± SD)	Kappa	
Aortic valves	4.73 ± 0.46	0.88	4.60 ± 0.47	0.76	—	—	0.045
Access route	4.57 ± 0.46	0.70	—	—	4.39 ± 0.48	0.89	0.014

Abbreviations: CT, computed tomography; CTA, CT angiography; SD, standard deviation; TAVI, transcatheter aortic valve implantation.

### 3.3 Diagnostic image quality

**FIGURE 4:**
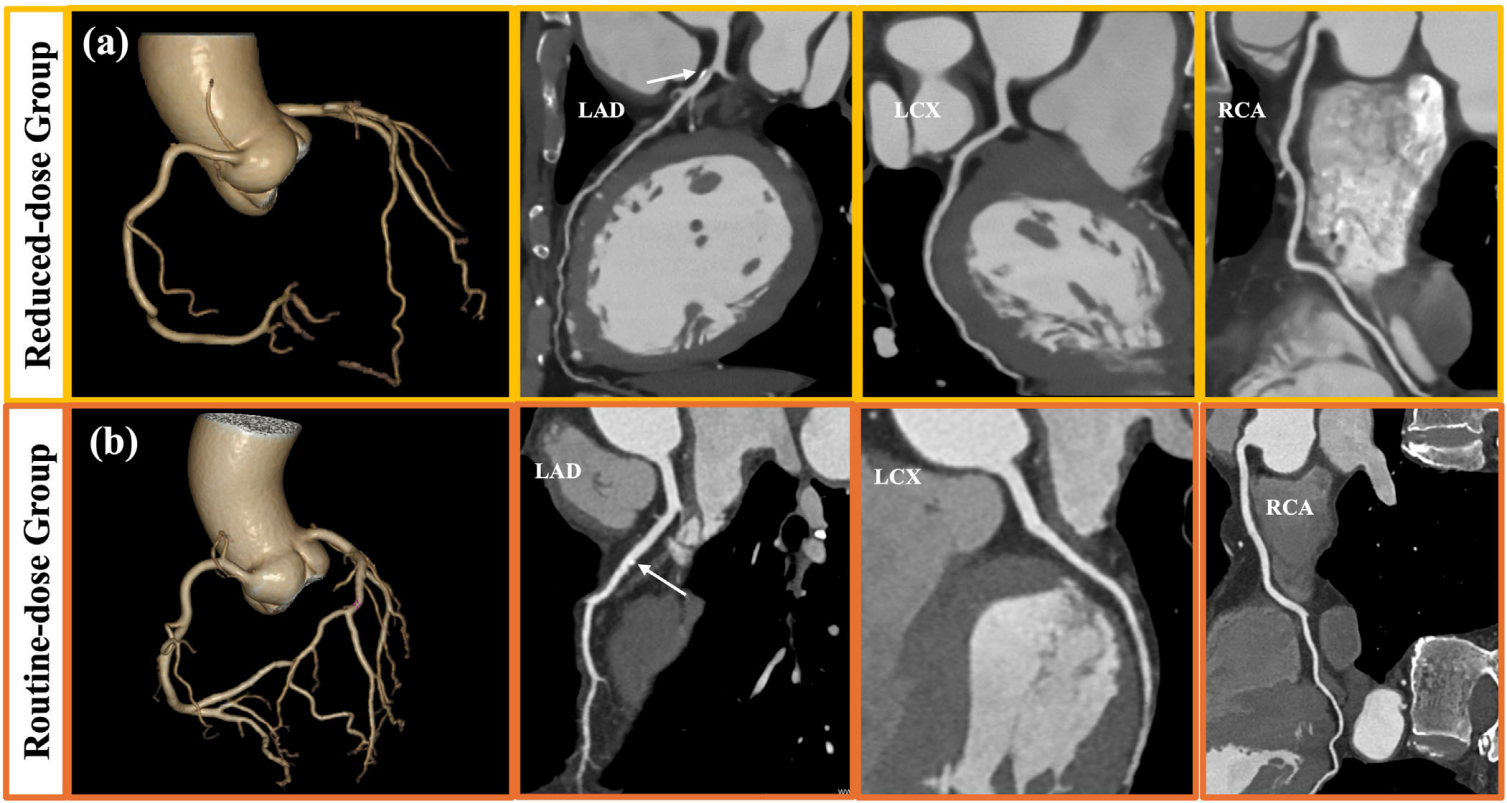
Comparison of the CCTA images between groups A and B. (a) the VR and CPR images of a 59‐year‐old woman (BMI, 24.44 kg m^−2^, HR, 52 bpm) in group A (reduced‐dose TAVI planning CT); (b) the VR and CPR images of a 65‐year‐old woman (BMI, 23 kg m^−2^, HR, 55 bpm) in group B (routine‐dose CCTA). Both patients were identified with calcified plaques (white arrow) on the LAD. CCTA, coronary CT angiography; CPR, curved planar reconstruction; CT, computed tomography; CTA, CT angiography; LAD, left anterior descending; LCX, left circumflex; RCA, right coronary artery; TAVI, transcatheter aortic valve implantation; VR, volume rendering.

A comparison of images of the coronary arteries in groups A and B is presented in Figure 4. The corresponding scores of diagnostic confidences as well as the inter‐observer agreement are listed in Table 3. No statistically significant difference was found between the two groups (all *p* > 0.05), suggesting comparable image quality in terms of diagnosing coronary arteries. On the aortic and iliac segments, diagnostic confidence as graded for group A was significantly higher than that for group C (*p* < 0.05), which might be owing to the noise suppression effect of the applied AIIR that led to a strong visual appearance on larger vessels. Overall, the inter‐observer agreement in terms of diagnostic image quality was moderate to excellent (0.70 ≤ κ ≤ 0.85).

**TABLE 3:** Diagnostic image quality scores.

	Group A Reduced‐dose TAVI planning CT		Group B Routine‐dose CCTA		Group C Routine‐dose aortic CTA		*p*
	Score (mean ± SD)	Kappa	Score (mean ± SD)	Kappa	Score (mean ± SD)	Kappa	
CCTA							
Left anterior descending	4.42 ± 0.55	0.77	4.44 ± 0.60	0.85	—	—	0.716
Left circumflex	4.54 ± 0.60	0.74	4.46 ± 0.66	0.75	—	—	0.454
Right coronary artery	4.19 ± 0.70	0.72	4.39 ± 0.52	0.81	—	—	0.097
Aortoiliac/aortic CTA							
Thoracic aorta	4.26 ± 0.44	0.74	—	—	4.03 ± 0.40	0.80	0.001
Abdominal aorta	4.92 ± 0.25	0.75	—	—	4.88 ± 0.31	0.70	0.276
Iliac artery	4.90 ± 0.29	0.84	—	—	4.69 ± 0.44	0.82	0.001
Femoral artery	4.95 ± 0.20	0.74	—	—	4.95 ± 0.20	0.85	0.816

Abbreviations: CCTA, coronary CT angiography; CT, computed tomography; CTA, CT angiography; SD, standard deviation; TAVI, transcatheter aortic valve implantation.

A total of 162 coronary artery vessels were identified with stenosis, including 37 with <50% stenosis and 34 with ≥50% stenosis in A and 61 with <50% stenosis and 30 with ≥50% stenosis in B. In subgroup analysis of the diagnostic confidence scores, no significant difference was observed between the two groups for those with <50% stenosis (4.34 vs. 4.44, *p* = 0.432) or with ≥50% stenosis (4.22 vs. 4.33, *p* = 0.955), suggesting a rather stable behavior of the DELTA algorithm in handling low‐dose images regardless of the characteristics of the lesion. For those with ≥50% stenosis, almost all received a score >3 in both groups, which was 31 of 34 (91%) in group A and 26 of 30 (87%) in group B. The inter‐observer agreement relating to the subgroup analysis was moderate to excellent (0.77 ≤ κ ≤ 0.88).

### 3.4 Objective image quality

**FIGURE 5:**
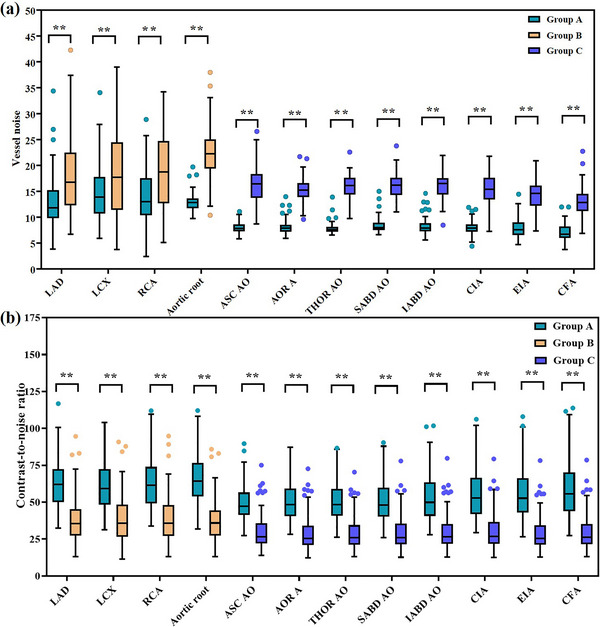
Box plot of image noise (a) and contrast‐noise‐ratio (b) measured at different sites. **: *p* < 0.001, statistically significant difference. Group A: reduced‐dose TAVI planning CT; Group B: routine‐dose CCTA; Group C: routine‐dose aortic CTA. CCTA, coronary CT angiography; CT, computed tomography; CTA, CT angiography; TAVI, transcatheter aortic valve implantation.

Figure 5 plots the image noise and CNR measured at different sites. For the coronary arteries, the image noise in group A was 21%–55% lower as compared to group B (all *p* < 0.001), resulting in a 63%–79% increase of the CNR (all *p* < 0.001). For the aortoiliac arteries, group A showed a 48%–53% reduction in noise as compared to group C (all *p* < 0.001), along with a significant increase in CNR (all *p* < 0.001) on the ascending aorta (79%), the aortic arch (90%), and the descending thoracic aorta (86%), as well as on the abdominal aorta and iliofemoral arteries (85%–114%). In terms of potential discrepancy between images acquired by the two parts of the TAVI planning CT, the two deep‐learning reconstruction algorithms seemed to have narrowed the difference and provided more consistent image appearance as compared to that between the routine CCTA and aortic CTA scans, which, for example, is indicated by noise measured on the aortic root and the ascending aorta.

## 4 DISCUSSION

This prospective study addressed the topic of reduced‐dose TAVI planning CT in three novel perspectives: it was investigated on a scanner model with 8‐cm detector, where both the CCTA part with retrospective ECG‐gated acquisition and the aortoiliac CTA part have to be acquired in helical mode; two different deep‐learning image reconstruction algorithms, one available for the ECG‐gated CCTA and one for the non‐gated aortoiliac CTA, were brought into use for dose reduction without causing discrepancy; the simulation approach based on routine aortic CTA patients have helped overcome the difficulty of lacking subjects at present without posing additional burden in radiation dose or taking the experimental risk on real cases of TAVI planning CT. Although the limit of dose reduction was not explored in this investigation, TAVI planning CT with proper dose control that represents >50% reduction of that reported on in‐kind scanner models has been proven feasible in the perspectives of both surgical planning and diagnosis.

Apart from the objective and subjective analysis included above, a comparison against TAVI planning CT with other scanners and protocols is possible through data reported in the literature. Compared with the study by Spagnolo et al.,^16^ which was performed on 4‐cm detector CT using retrospective ECG‐gated scan of the thorax and the abdomen with 80–100 kVp and 450–550 mA, our results showed remarkable noise reduction at substantially reduced dose (59% reduction, 8.22 ± 0.83 mSv vs. 20.2 ± 4.6 mSv) through the application of the deep‐learning reconstruction algorithm. Fogante et al.^12^ tested a dual‐source scanner and prospective ECG‐gated ultra‐high pitch acquisition in TAVI planning CT; only the CNR data were made available, where the mean ranged from 14.0 ± 2.7 to 17.1 ± 3.6 among the measured ROIs for CCTA and from 14.2 ± 4.2 to 19.0 ± 5.8 for aortoiliac CTA. Unfortunately, a straightforward comparison of the CNR is not feasible, because the background used in the calculation was defined on different regions where the CT number could vary a lot.

The deep‐learning reconstruction algorithms utilized in this study demonstrate significant noise suppression and higher contrast resolution compared to other deep‐learning reconstruction algorithms. Compared with the study by Heinrich et al.,^27^ where deep‐learning reconstruction was used for TAVI planning CT on wide‐detector CT at 100 kVp, our results showed greater noise suppression on the aortoiliac CTA images, where the median was 7.80 HU versus 22.00 HU on the ascending aorta, 7.60 HU versus 23.00 HU on the thoracic descending aorta, 7.90 HU versus 20.00 HU on the abdominal aorta, and 7.70 HU versus 18.00 HU on the pelvic arteries. In the study of Zhang et al.,^15^ where the deep‐learning reconstruction was used in combination with a low‐dose (80 kVp) and low‐contrast dosage (45.28 mL) protocol for TAVI planning CT, the same CNR formula as in our study was employed for assessing the aortoiliac CTA images. Our data demonstrated a remarkably higher contrast resolution, where the mean CNR was 48.31 versus 26.02 on the aortic arch, 47.18 versus 25.40 on the ascending aorta, and 48.44 versus 26.91 on the descending aorta.

Next to the pros and cons of the simulation approach, four limitations must be noted for the present work: First, the enrolled subjects were relatively younger than the population typically considered for TAVI. This could have been improved by including more patients of advanced age (e.g., >80 y), where more cardiovascular abnormalities might be present and make the analysis more relevant to the scenario of TAVI planning CT; Second, the evaluation of image quality relied on subjective and objective metrics without assessing the actual diagnostic accuracy, for example, detecting and characterizing stenosis on coronary arteries. This was due to the lack of a gold standard, which remains an interesting point of investigation as more follow‐up information or data from other examinations becomes available; Third, tube current modulation was not utilized during the acquisition of CCTA. Integrating it with advanced image reconstruction algorithms could have resulted in greater dose reduction, especially considering the variability in patient sizes. Finally, validation with real‐world TAVI planning cases instead of simulation and optimizing the dosage of contrast agent on top of the present investigation might both be further steps worth exploration.

## 5 CONCLUSION

It is feasible to carry out TAVI planning CT on 8‐cm detector scanner models with proper dose control by using the deep‐learning reconstruction algorithms. Though tested only with simulated cases for one scanner model, the demonstrated feasibility remains translational to others with single‐source and medium‐size detectors (4–8 cm), showing promise of implementing TAVI planning CT in imaging settings that are more commonly accessible.

## AUTHOR CONTRIBUTIONS

Qiang Shao, Yongxia Zhou, and Yonghong Du conceived of the project. Qiang Shao, Yongxia Zhou, Kunyao Li, Zhaojun Ding, Rong Zhou, Jiamo Zhang, Xiaohong Zheng, and Yonghong Du designed the study and performed the measurements. Qiang Shao, Yongxia Zhou, and Jing Li performed the data analysis. Qiang Shao and Yongxia Zhou drafted the manuscript. Yongxia Zhou and Jiamo Zhang arranged funding. Qiang Shao, Yongxia Zhou, Jing Li, Guozhi Zhang, and Yonghong Du revised the manuscript. All authors approved the manuscript version to be published.

## CONFLICT OF INTEREST STATEMENT

Two of the authors (J.L. and G.Z.) are scientific researchers with the United Imaging Healthcare. The remaining authors declare no relationships with any companies.

## ETHIC STATEMENT

This work was carried out under Institutional Review Board approval number 2024EC0005.

## Data Availability

Authors will share data upon request to the corresponding author.

## References

[1] Cahill TJ , Chen M , Hayashida K , et al. Transcatheter aortic valve implantation: current status and future perspectives. Eur Heart J. 2018;39(28):2625‐2634. 29718148 10.1093/eurheartj/ehy244

[2] Leon MB , Smith CR , Mack MJ , et al. Transcatheter or surgical aortic‐valve replacement in intermediate‐risk patients. N Engl J Med. 2016;374(17):1609‐1620. 27040324 10.1056/NEJMoa1514616

[3] Smith CR , Leon MB , Mack MJ , et al. Transcatheter versus surgical aortic‐valve replacement in high‐risk patients. N Engl J Med. 2011;364(23):2187‐2198. 21639811 10.1056/NEJMoa1103510

[4] Mack MJ , Leon MB , Thourani VH , et al. Transcatheter aortic‐valve replacement with a balloon‐expandable valve in low‐risk patients. N Engl J Med. 2019;380(18):1695‐1705. 30883058 10.1056/NEJMoa1814052

[5] Blanke P , Schoepf UJ , Leipsic JA . CT in transcatheter aortic valve replacement. Radiology. 2013;269(3):650‐669. 24261496 10.1148/radiol.13120696

[6] Leipsic J , Gurvitch R , LaBounty TM , et al. Multidetector computed tomography in transcatheter aortic valve implantation. JACC: Cardiovasc Imaging. 2011;4(4):416‐429. 21492818 10.1016/j.jcmg.2011.01.014

[7] Blanke P , Weir‐McCall JR , Achenbach S , et al. Computed tomography imaging in the context of transcatheter aortic valve implantation (TAVI)/transcatheter aortic valve replacement (TAVR). JACC: Cardiovasc Imaging. 2019;12(1):1‐24. 30621986 10.1016/j.jcmg.2018.12.003

[8] Dankerl P , Hammon M , Seuss H , et al. Computer‐aided evaluation of low‐dose and low‐contrast agent third‐generation dual‐source CT angiography prior to transcatheter aortic valve implantation (TAVI). Int J Comput Assist Radiol Surg. 2017;12(5):795‐802. 27604759 10.1007/s11548-016-1470-8

[9] Felmly LM , De Cecco CN , Schoepf UJ , et al. Low contrast medium‐volume third‐generation dual‐source computed tomography angiography for transcatheter aortic valve replacement planning. Eur Radiol. 2017;27(5):1944‐1953. 27553939 10.1007/s00330-016-4537-6

[10] Mangold S , De Cecco CN , Schoepf UJ , et al. CT angiography for planning transcatheter aortic valve replacement using automated tube voltage selection: image quality and radiation exposure. Eur J Radiol. 2017;86:276‐283. 28027760 10.1016/j.ejrad.2016.11.023

[11] Schicchi N , Fogante M , Pirani PE , et al. Third generation dual source CT with ultra‐high pitch protocol for TAVI planning and coronary tree assessment: feasibility, image quality and diagnostic performance. Eur J Radiol. 2020;122:108749. 31759224 10.1016/j.ejrad.2019.108749

[12] Fogante M , Esposto Pirani P , Cela F , et al. Ultra‐low radiation dose and contrast volume CT protocol and TAVI‐CT score for TAVI planning and outcome. Br J Radiol. 2023;96(1148):1‐8. 10.1259/bjr.20221026 PMC10392642 37183830

[13] Zhang Y , Li Z , You Y , Peng L , Li J , Shuai T . Image quality and diagnostic performance evaluation in transcatheter aortic valve implantation candidates with atrial fibrillation using a whole‐heart coverage CT scanner. Eur Radiol. 2022;32(2):1034‐1043. 34338842 10.1007/s00330-021-08187-z

[14] Cour A , Burel J , Garnier M , Durand E , Demeyere M , Dacher J‐N . CT annulus sizing prior to transcatheter aortic valve replacement (TAVR): evaluation of free‐breathing versus breath‐holding acquisition. Eur Radiol. 2023;33(12):8521‐8527. 37470824 10.1007/s00330-023-09913-5

[15] Zhang Y , Liu Z , Cheng Y , et al. Deep learning image reconstruction for transcatheter aortic valve implantation planning: image quality, diagnostic performance, contrast volume and radiation dose assessment. Acad Radiol. 2024;31(6):2268‐2280. 38472024 10.1016/j.acra.2024.02.026

[16] Spagnolo P , Giglio M , Di Marco D , et al. Feasibility of ultra‐low contrast 64‐slice computed tomography angiography before transcatheter aortic valve implantation: a real‐world experience. Eur Heart J Cardiovasc Imaging. 2016;17(1):24‐33. 26160397 10.1093/ehjci/jev175

[17] DELTA: Deep Learning Based CT Reconstruction (Version 01), Technical White Paper. United Imaging Healthcare; 2020. https://www.united‐imaging.com

[18] AIIR‐the World's Pioneering CT Image Reconstruction Technology (Version 01), Technical White Paper. United Imaging Healthcare; 2023. https://www.united‐imaging.com

[19] You Y , Zhong S , Zhang G , et al. Exploring the low‐dose limit for focal hepatic lesion detection with a deep learning‐based CT reconstruction algorithm: a simulation study on patient images. J Imaging Inform Med. 2024;37:2089‐2098. doi:10.1007/s10278-024-01080-3 38502435 PMC11522246

[20] Gong H , Peng L , Du X , et al. Artificial intelligence iterative reconstruction in computed tomography angiography: an evaluation on pulmonary arteries and aorta with routine dose settings. J Comput Assist Tomogr. 2024;48(2):244‐250. 37657068 10.1097/RCT.0000000000001542

[21] Bai K , Wang T , Zhang G , et al. Improving intracranial aneurysms image quality and diagnostic confidence with deep learning reconstruction in craniocervical CT angiography. Acta Radiol. 2024;65(8):913‐921. 38839094 10.1177/02841851241258220

[22] Li J , Zhu J , Zou Y , et al. Diagnostic CT of colorectal cancer with artificial intelligence iterative reconstruction: a clinical evaluation. Eur J Radiol. 2024;171:111301. 38237522 10.1016/j.ejrad.2024.111301

[23] Yang L , Liu H , Han J , et al. Ultra‐low‐dose CT lung screening with artificial intelligence iterative reconstruction: evaluation via automatic nodule‐detection software. Clin Radiol. 2023;78(7):525‐531. 36948944 10.1016/j.crad.2023.01.006

[24] Li W , You Y , Zhong S , et al. Image quality assessment of artificial intelligence iterative reconstruction for low dose aortic CTA: a feasibility study of 70 kVp and reduced contrast medium volume. Eur J Radiol. 2022;149:110221. 35196615 10.1016/j.ejrad.2022.110221

[25] Zhuo L , Xu S , Zhang G , et al. Ultralow dose coronary calcium scoring CT at reduced tube voltage and current by using deep learning image reconstruction. Eur J Radiol. 2024;181:111742. 39321657 10.1016/j.ejrad.2024.111742

[26] Soon J , Sulaiman N , Park JK , et al. The effect of a whole heart motion‐correction algorithm on CT image quality and measurement reproducibility in Pre‐TAVR aortic annulus evaluation. J Cardiovasc Comput Tomogr. 2016;10(5):386‐390. 27576115 10.1016/j.jcct.2016.08.001

[27] Heinrich A , Yucel S , Bottcher B , et al. Improved image quality in transcatheter aortic valve implantation planning CT using deep learning‐based image reconstruction. Quant Imaging Med Surg. 2022;13(2):970‐981. 36819291 10.21037/qims-22-639 PMC9929406

